# Bayesian Modeling of Prion Disease Dynamics in Mule Deer Using Population Monitoring and Capture-Recapture Data

**DOI:** 10.1371/journal.pone.0140687

**Published:** 2015-10-28

**Authors:** Chris Geremia, Michael W. Miller, Jennifer A. Hoeting, Michael F. Antolin, N. Thompson Hobbs

**Affiliations:** 1 Natural Resource Ecology Laboratory, Department of Ecosystem Science and Sustainability and Graduate Degree Program in Ecology, Colorado State University, Fort Collins, Colorado, United States of America; 2 Wildlife Health Program, Colorado Division of Parks and Wildlife, Fort Collins, Colorado, Colorado, United States of America; 3 Department of Statistics, Colorado State University, Fort Collins, Colorado, United States of America; 4 Department of Biology, Colorado State University, Fort Collins, Colorado, United States of America; Colorado State University, College of Veterinary Medicine and Biomedical Sciences, UNITED STATES

## Abstract

Epidemics of chronic wasting disease (CWD) of North American *Cervidae* have potential to harm ecosystems and economies. We studied a migratory population of mule deer (*Odocoileus hemionus*) affected by CWD for at least three decades using a Bayesian framework to integrate matrix population and disease models with long-term monitoring data and detailed process-level studies. We hypothesized CWD prevalence would be stable or increase between two observation periods during the late 1990s and after 2010, with higher CWD prevalence making deer population decline more likely. The weight of evidence suggested a reduction in the CWD outbreak over time, perhaps in response to intervening harvest-mediated population reductions. Disease effects on deer population growth under current conditions were subtle with a 72% chance that CWD depressed population growth. With CWD, we forecasted a growth rate near one and largely stable deer population. Disease effects appear to be moderated by timing of infection, prolonged disease course, and locally variable infection. Long-term outcomes will depend heavily on whether current conditions hold and high prevalence remains a localized phenomenon.

## Introduction

Epizootics can affect the health and stability of host populations, ecosystems, and human economies [1–2]. Rinderpest provides a dramatic historic example of episodic viral outbreaks leading to sharp declines in buffalo (*Syncerus caffer*) and wildebeest (*Connochates taurinus*) populations in the Serengeti, with cascading effects on predator-prey and grassland dynamics, as well as food production [3]. More recently, but no less dramatically, marine epizootics continue to alter community structure and ecosystem processes by reducing reef-building corral species [4–6] and the spread of chytrid fungus coincided with a worldwide amphibian decline [7].

Less dramatic effects of an epizootic on ecosystems almost certainly occur, but can be more challenging to observe and quantify when the only readily available data are collected over the relatively brief time spans of most field studies. Studies of epidemics over larger geographic areas and longer time horizons are often needed to better understand the ultimate course of such epidemics and the fate of affected host populations. But disease surveillance and systematic sampling is seldom initiated without *a priori* evidence suggesting an imminent catastrophe. It follows that analytical approaches exploiting relatively simple and available repeated measurements of key population and epidemic parameters could afford critical insights into the ecological consequences of epidemics that play out over protracted time scales.

Chronic wasting disease (CWD) exemplifies the foregoing challenges and opportunities. Caused by prions (misfolded proteins) and now occurring naturally in North American cervids [8–9], this emergent wildlife disease has raised concern because infections are fatal, the infectious agent can be transmitted among species within the deer family, and control strategies remain elusive [9–11]. Infectious individuals shed prions through feces, saliva, and urine [12–14]. Transmission can occur directly between animals or indirectly from a reservoir of shed prions that persist in the environment [15–17].

Empirical studies show that CWD lowers survival of infected deer and can depress population growth rates locally when infection prevalence becomes sufficiently high [18–20]. Sustained declines in cervid abundance over larger areas may harm ecosystems and human economies because herbivory by deer affects plant communities, deer are primary prey for large carnivores, and deer serve as a food and recreational resource to people. However, simulation models offer widely varied predictions on the consequence of CWD for affected populations [21–24]. Modeled epizootic outcomes have ranged from limited population decline and sustained low disease prevalence to local extinction within decades of disease introduction.

**Fig 1:**
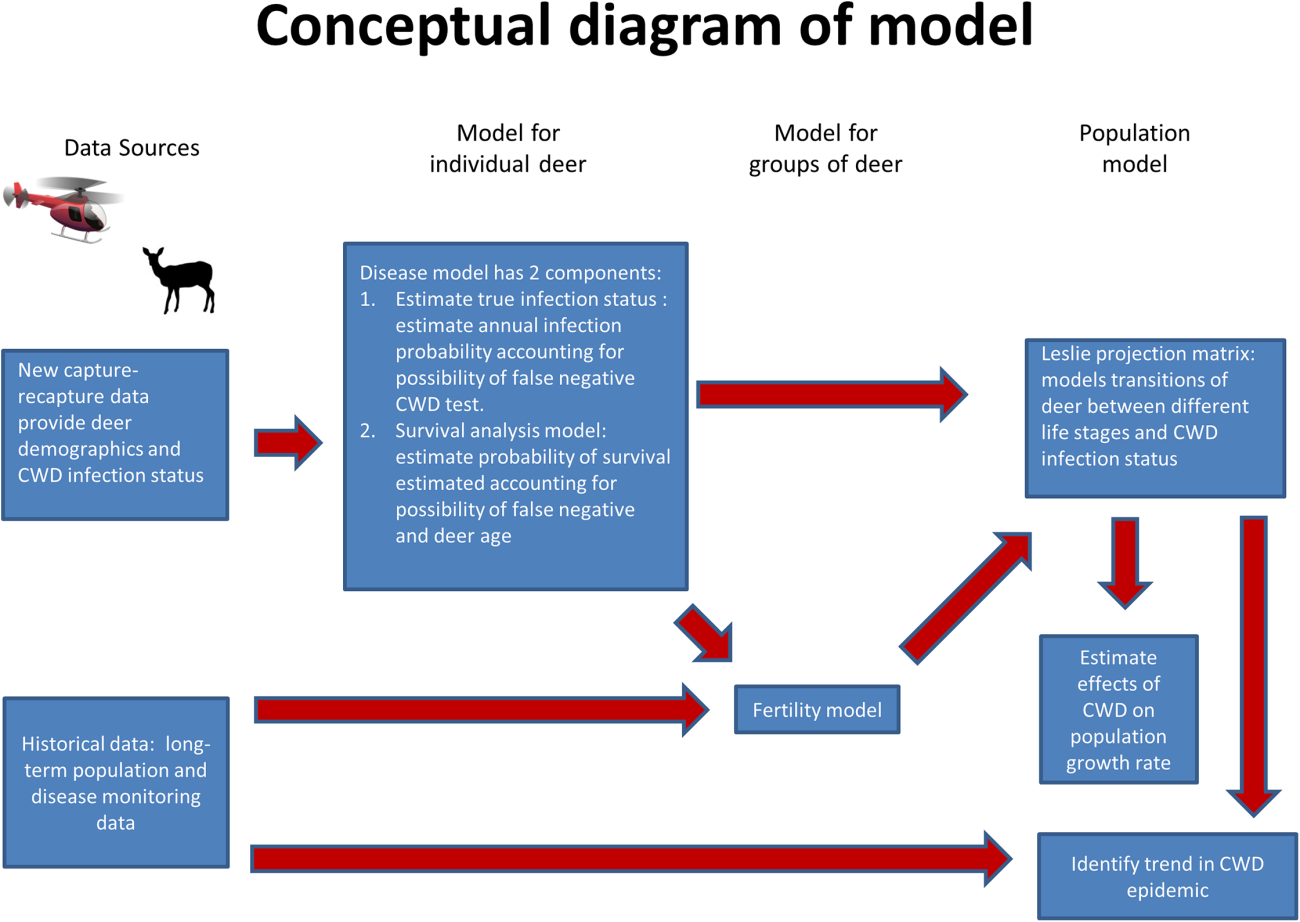
Hierarchical Bayesian framework used to integrate a matrix population and disease model with field data. We assimilated a model with long-term monitoring and capture-recapture data to forecast the effect of CWD on deer population growth, forecast short-term CWD prevalence, and assess how the epidemic is changing.

In this study, we used a Bayesian framework and a matrix model of demographic and disease processes to integrate long-term monitoring data and detailed process-level studies to gain insight into CWD dynamics in mule deer (*Odocoileus hemionus*) herds in the Laramie Foothills of north-central Colorado and southern Wyoming, U.S.A (Fig 1). This population was among those where CWD was first detected in free-ranging mule deer in 1985 [25]. The disease may have been present in the population for two decades before it was detected [24].

We created a single sex, Leslie projection matrix to describe a mule deer population chronically infected with CWD. The projection matrix was composed of survival, fertility, and infection parameters. We then estimated values of projection matrix parameters from recent capture-recapture data and long-term helicopter survey data using a hierarchical Bayesian model. Monte Carlo methods and eigen-analysis were used to forecast the growth rate of the population with and without CWD, and measure the magnitude of the effect of CWD on population growth.

Nonlinear infection probability is a hallmark of disease transmission because the per capita rate of new infections changes with both the number/density of infected individuals and, in the case of CWD, with abundance of infectious prions in the environment. However, we were not able to observe the abundance of infectious prions in the environment at the scale of our research [26]. Therefore, we used our Leslie model, a linear model, to provide short-term forecasts of CWD prevalence under the survival, fertility, and infection conditions we measured. This allowed us to compare forecasted prevalence to what was observed historically (1997−2002 and 2010−2011) and determine whether the epidemic is changing.

## Results

### Deer Abundance

**Fig 2:**
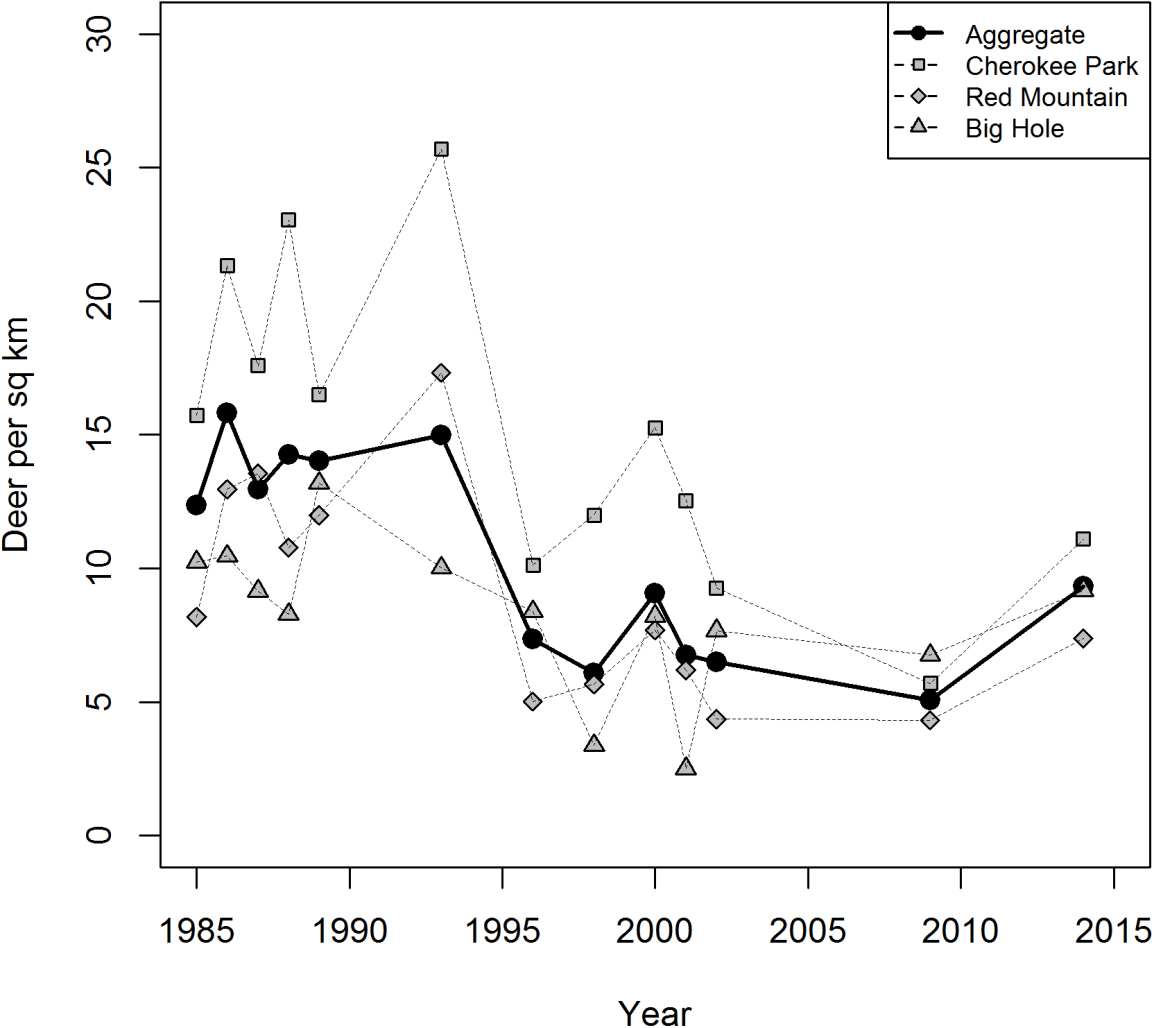
Average density of the deer population and individual wintering subpopulations. Average deer density (km^-2^) for the overall study area (in aggregate) and for the Big Hole, Cherokee Park, and Red Mountain wintering subpopulations during 1985–2014.

We studied a deer population that could be delineated into four wintering subpopulations known to have limited spatial overlap. Hereafter, we refer to the entire population as “in aggregate” and the individual subpopulations as “Big Hole,” “Red Mountain,” “Campbell Valley,” and “Cherokee Park”. The approximate size of wintering areas were 196 km^2^ for Big Hole, 113 km^2^ for Cherokee Park, 60 km^2^ for Campbell Valley, and 199 km^2^ for Red Mountain. In 1985, average deer densities were 12.35 deer/km^2^ in aggregate, 10.24 deer/km^2^ in Big Hole, 15.72 deer/km^2^ in Cherokee Park, and 8.18 deer/km^2^ in Red Mountain (data were unavailable in Campbell Valley). Since then, the population was subject to a series of changing management policies that initially prioritized male harvest (1990−2000), then reduced deer abundance by increasing female harvest (2001−2006), and then promoted population increase by limiting harvest (2006−2014). In response to management (and perhaps other factors), average deer densities had declined to 5.07 deer/km^2^ in aggregate, 6.75 deer/km^2^ in Big Hole, 5.70 deer/km^2^ in Cherokee Park, and 4.32 deer/km^2^ in Red Mountain by the beginning of our capture-recapture study (Fig 2). During our capture-recapture study, the Big Hole and Red Mountain subpopulations were stable, with some indication of growth in the aggregate population that largely resulted from an increasing Cherokee Park subpopulation (Fig 2).

### Infection Probability

During our capture-recapture study, we monitored 217 female deer with individuals followed for up to five years and observations totaling 608 animal years. We assigned 67 deer to the Big Hole herd, 39 to Cherokee Park, 30 to Campbell Valley, and 81 to Red Mountain based on their centers of activity. On average, we recaptured 85% of surviving deer each winter.

Twenty-two of the deer in our capture-recapture sample were observed to be infected with CWD. Seven of these entered our research as infected and 15 became infected while under study. Ages (in years) of conversion from susceptible to infected were 1.5 (1 deer; “1”), 2.5 (1), 3.5 (2), 4.5 (2), 5.5 (2), 6.5 (4), 7.5 (2), and 8.5 (1). New infections were not observed in deer >8.5 years old.

**Fig 3:**
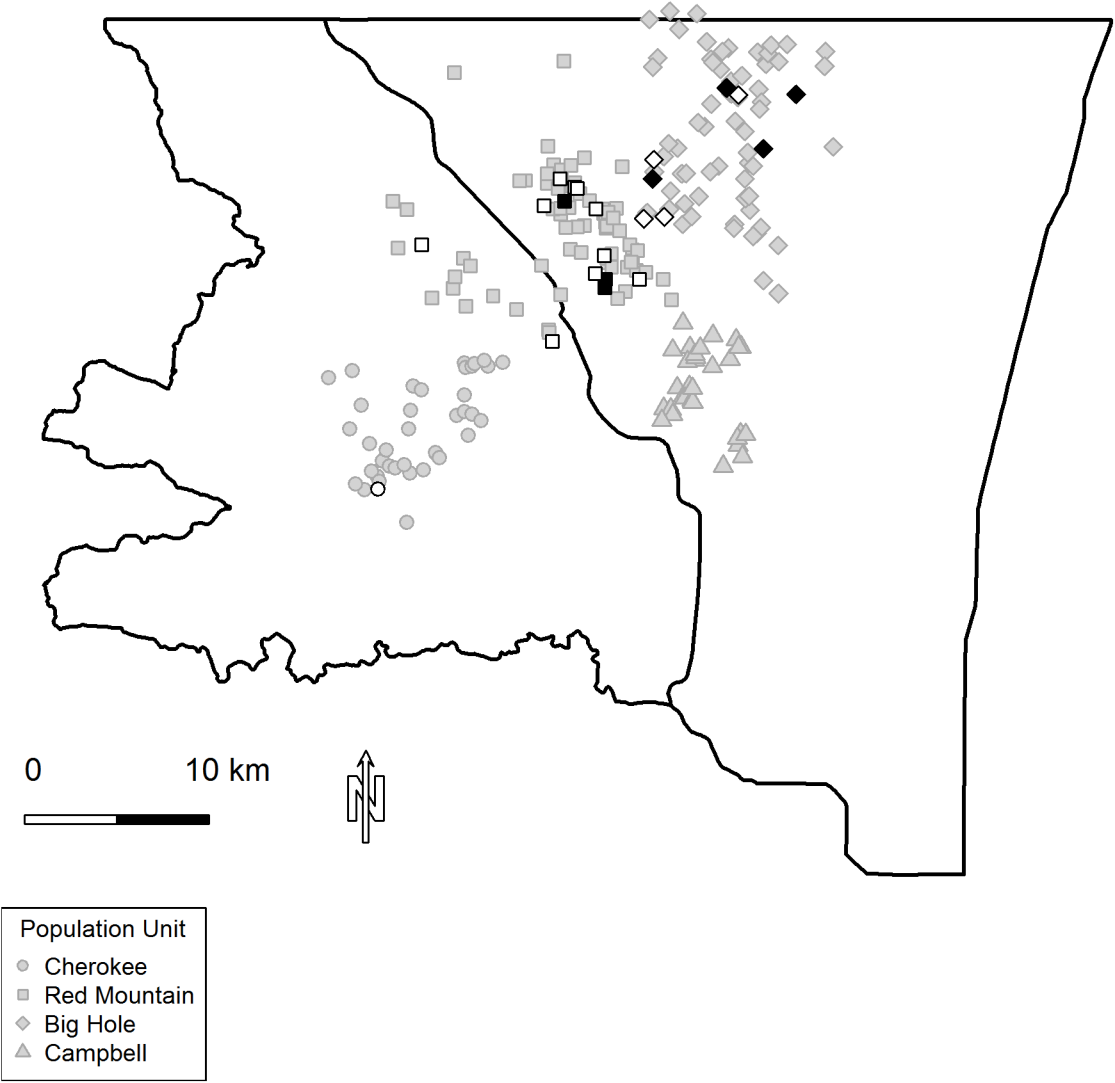
Spatial distribution of CWD infected deer. Median winter locations of female mule deer studied during 2010–2014. Deer that entered (black) or converted (white) to CWD positive were mostly located in a 50 km^2^ region in the center of the study area.

CWD infection probability was 0.04 (0.02−0.06; equal-tailed credible interval; Table 1). Spatial differences in infection probability among wintering subpopulations were striking. New infections were spatially localized, with all but one occurring in the Red Mountain or Big Hole herds. Nearly 70% of new infections occurred within a ~50 km^2^ area (Fig 3), and new infections were observed in this focus each year of the study. Infection probability among wintering herds varied by nearly an order of magnitude, with highest annual infection probability in Red Mountain 0.07 (0.03−0.12). Deer occupying wintering areas less than 30 km away from this apparent 'hot spot' had annual infection probabilities <0.01 (Table 1).

**Table 1:** Estimates of annual CWD infection probability.

		Posterior Distribution Quantiles		
**Spatial Extent**	**Mean**	**0.025%**	**0.500%**	**0.975%**
**Red Mountain**	0.07	0.03	0.06	0.12
**Big Hole**	0.04	0.01	0.04	0.09
**Campbell Valley**	<0.01	<0.01	<0.01	0.03
**Cherokee Park**	0.02	<0.01	0.01	0.06
**in Aggregate**	0.04	0.02	0.04	0.07

In aggregate refers to the entire population. Red Mountain, Big Hole, Campbell Valley and Cherokee Park are wintering subpopulations.

CWD status was determined from immunohistochemistry staining of rectal-anal mucosa associated lymphoid tissues. In a disease test, up to several lymphoid follicles are observed in collected tissue and CWD infection is tested for in each follicle. The chance that a single follicle tested positive in an infected deer was 0.56 (*π* = 0.56, 0.51−0.61), meaning that testing at least five follicles was necessary to assure a 95% accurate test. The average follicle count of tissue samples among tests was 14.2 follicles–suggesting tests correctly identified infected deer.

### Survival and Reproduction

**Fig 4:**
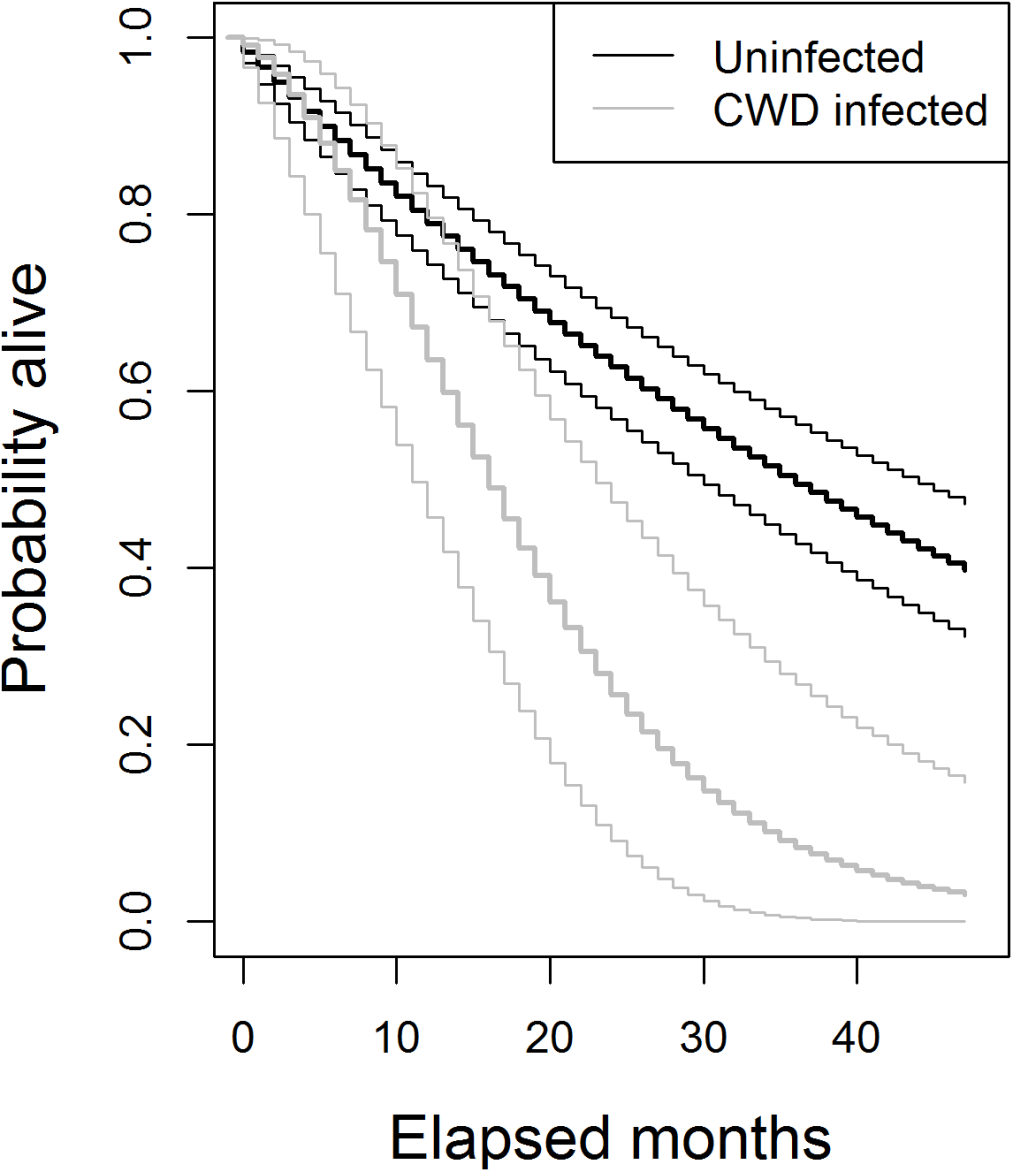
Deer survival after testing positive for CWD. Median (bold) and 95% credible intervals of probabilities of surviving the given number of additional months.

After initial detection of CWD, average deer lifespan was 410 days (range = 41−1,016). Two infected deer survived to the end of our study and lived for more than 700 days. The hazard rate (e.g., chance of mortality) progressively increased after an individual became infected (*α*_2_ = 1.96, 1.18−2.75). This equated to a 0.67 (0.49−0.83) chance of surviving one more year after infection, but only a 0.05 (<0.01–0.23) chance of surviving three additional years (Fig 4).

Survival probability of uninfected deer, averaged across ages and wintering areas, was 0.82 (0.77−0.86, Table 2). Hazards were higher for deer that were older at the time of first capture–survival of uninfected deer decreased with age from 0.87 (0.82−0.91) at 1.5 years old to 0.62 (0.44−0.77) at 10.5 years old. There was some indication that uninfected deer survival varied among wintering subpopulations. Across ages, annual survival probabilities were 0.81 in Campbell Valley (0.69−0.90), 0.87 in Cherokee Park (0.79−0.94), 0.74 in Big Hole (0.65−0.83), and 0.83 in Red Mountain (0.76−0.89).

**Table 2:** Estimates of hazard model parameters for uninfected and CWD infected deer.

		Posterior Distribution Quantiles		
Parameter	Mean	0.025%	0.500%	0.975%
*α*_1_ ^1^	1.09	0.92	1.09	1.30
*α*_2_ ^2^	1.96	1.18	1.96	2.75
*λ*_1_ ^1^	0.013	0.004	0.011	0.028
*λ*_2_ ^2^	0.005	<0.001	0.002	0.025
*β*_1_ ^3^	1.15	-0.09	1.17	2.28
*β*_2_ ^4^	-0.30	-1.11	-0.28	0.42
*β*_3_ ^5^	-0.75	-1.56	-0.74	0.02
*β*_4_ ^6^	-0.46	-1.09	-0.45	0.14

^1^ Hazard model parameters for uninfected deer: *α* affects the rate that hazards increased over time with values greater than one indicating increasing hazards; *λ* is the monthly hazard rate.

^2^ Hazard model parameters for CWD infected deer.

^3^ Age effects on hazards: values greater than zero show increased hazards.

^4–6^ Wintering subpopulation effects on hazards: values less than zero show decreased hazards relative to the Big Hole subpopulation—^4^ represents Campbell Valley—^5^ represents Cherokee Park—^6^ represents Red Mountain.

One hundred and fourteen deer died of natural causes (this excludes two deer harvested by hunters and eleven deaths from capture and handling). Eighty-one (71%) deaths were from unknown natural causes due to insufficient carcass material. Four (4%) deaths were directly attributed to CWD, but eight infected deer classified as unknown also may have succumbed to CWD based on timing and field observations. Death was attributed to predation in 23 cases (20%), and based on site characteristics 22 were killed by mountain lions. Two (1%) deaths were attributed to winter starvation and four (4%) to vehicle collision.

Recruitment was defined as the number of female fawns produced per female that survived from birth until winter census. CWD infection was not believed to affect recruitment [19]. Overall, each female produced 0.27 (0.22−0.32) female fawns that survived until winter census. There was some variation among wintering subpopulations: 0.26 (0.20−0.32) in Big Hole, 0.23 (0.12−0.37) in Cherokee Park, and 0.21 (0.13−0.28) in Red Mountain (data were unavailable in Campbell Valley).

### Population Growth Rate

**Fig 5:**
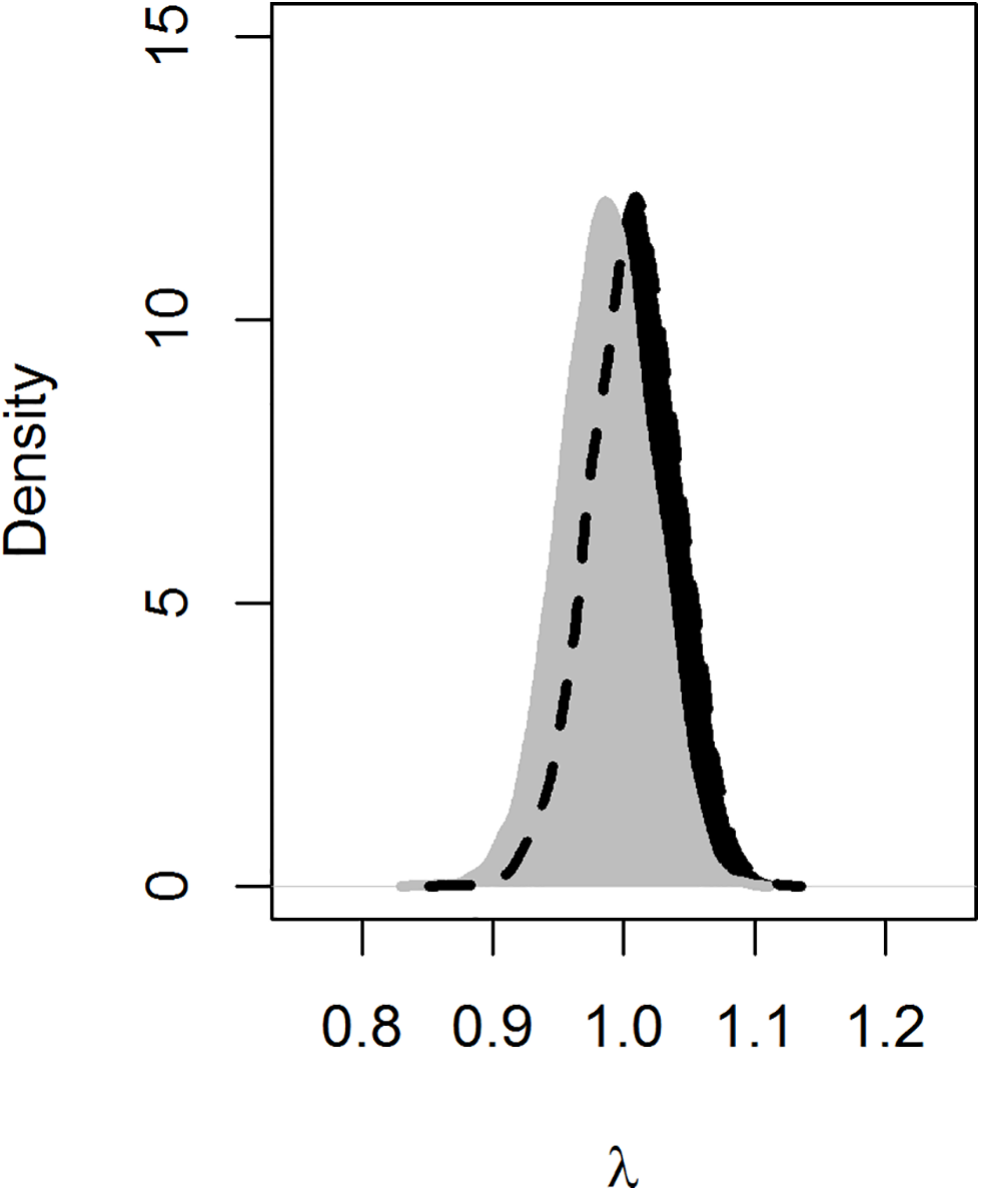
Estimates of population growth rates with and without CWD. Population growth rates using survival, fertility, and infection estimates aggregated among wintering subpopulations with (gray) and without (black) CWD. The amount that the gray polygon is shifted to the left towards zero shows the direct contribution of CWD to lowering growth rate under the conditions observed.

The findings on infection, survival, and reproduction described above were used as inputs in a Leslie matrix model (S1 Table) to estimate the effect of CWD on population growth rate. Aggregated among wintering subpopulations, the posterior distribution of population growth rates overlapped between populations with CWD (*λ*_CWD_ = 0.99; 0.92−1.05) and without CWD (*λ*_FREE_ = 1.01; 0.95−1.07, S1 Table). The posterior distribution of the difference in overall population growth rates averaged −0.02 (−0.11−0.07). This equated to a 72% chance that CWD lowered population growth rate (Fig 5). We forecasted similar chances of population growth or decline provided conditions remained as measured. Helicopter surveys suggested increases in deer density (in aggregate) during the timespan of the capture-recapture research (Fig 2).

The magnitude of CWD effects on population growth rate differed among the wintering subpopulations. Disease effects were strongest in Red Mountain with a 93% chance of local decline (*λ*_CWD_ = 0.93, 0.83−1.03) and 73% chance that population growth rate was lower with CWD. In Big Hole, local decline was the more likely outcome regardless of CWD, but CWD increased the chance of decline (Table 3). In contrast, we found virtually no impact of CWD on population growth rate in Cherokee Park or Campbell Valley where infections were rare (Table 3). Moreover, forecasted population growth rates without CWD were higher in Cherokee Park and Campbell Valley compared to Red Mountain and Big Hole. In agreement with these findings, helicopter surveys detected limited change in deer density over the past 20 years in the Big Hole and Red Mountain subpopulations. Increasing deer density was observed in Cherokee Park–a subpopulation with low CWD prevalence.

**Table 3:** Estimates of subpopulation growth rates with and without CWD.

			Posterior Distribution Quantiles		
Spatial Extent	*λ*	Mean	0.025%	0.500%	0.975%
**Red Mountain**	CWD	0.93	0.83	0.93	1.03
	No Disease	0.97	0.87	0.97	1.07
**Big Hole**	CWD	0.92	0.81	0.92	1.02
	No Disease	0.94	0.83	0.94	1.04
**Campbell Valley**	CWD	1.00	0.87	1.00	1.11
	No Disease	1.00	0.87	1.00	1.11
**Cherokee Park**	CWD	1.01	0.88	1.01	1.15
	No Disease	1.03	0.89	1.02	1.16

*λ* is the subpopulation growth rate, ‘CWD’ is the subpopulation growth rate under the disease conditions we measured, and ‘No Disease’ is the subpopulation growth rate in the absence of CWD.

### CWD Prevalence

Surveillance conducted during 1997–2002 indicated overall CWD prevalence in female deer >1 year old was 0.08 (0.06−0.11, Table 4). CWD prevalence among subpopulations was 0.17 (0.07−0.31; n = 33) in Big Hole, 0.11 (0.01−0.29; n = 16) in Campbell Valley, 0.07 (0.04−0.10; n = 213) in Cherokee Park, and 0.14 (0.08−0.21; n = 103) in Red Mountain (Table 4). Contemporary CWD prevalence during 2010–11 measured from our capture-recapture female deer at the time of first handling was lower: 0.04 (0.02−0.07) overall, 0.07 (0.03−0.15) in Big Hole, 0.03 (<0.01–0.12) in Campbell Valley, 0.03 (<0.01−0.09) in Cherokee Park, and 0.06 (0.02−0.13) in Red Mountain (Table 4).

**Table 4:** Female deer CWD test results.

Spatial Extent	1997–2002^1^	2010–2011^2^
Aggregate Population	34 (444)	8^3^ (210^4^)
Big Hole	5 (33)	4 (65)
Campbell Valley	1 (16)	0 (29)
Cherokee Park	13 (213)	0 (38)
Red Mountain	14 (103)	4 (78)

Historic CWD tests observed at spatial extents where numbers of positive tests are reported and numbers in parentheses show total tests for each year. CWD test results were limited to female deer classified as > 1 year of age by observers.

^1^ CWD status determined from immunohistochemistry exam retropharyngeal lymph node and tonsil tissue of harvested deer

^2^ CWD status determined from immunohistochemistry exam rectal-mucosa associated lymphatic tissue of helicopter captured deer

^3^ One individual was 1.5 years old and classified as becoming infected under study in the manuscript text

^4^ Seven additional deer were not included in this total that were fawns when initially captured that did not survive future recapture and testing

**Fig 6:**
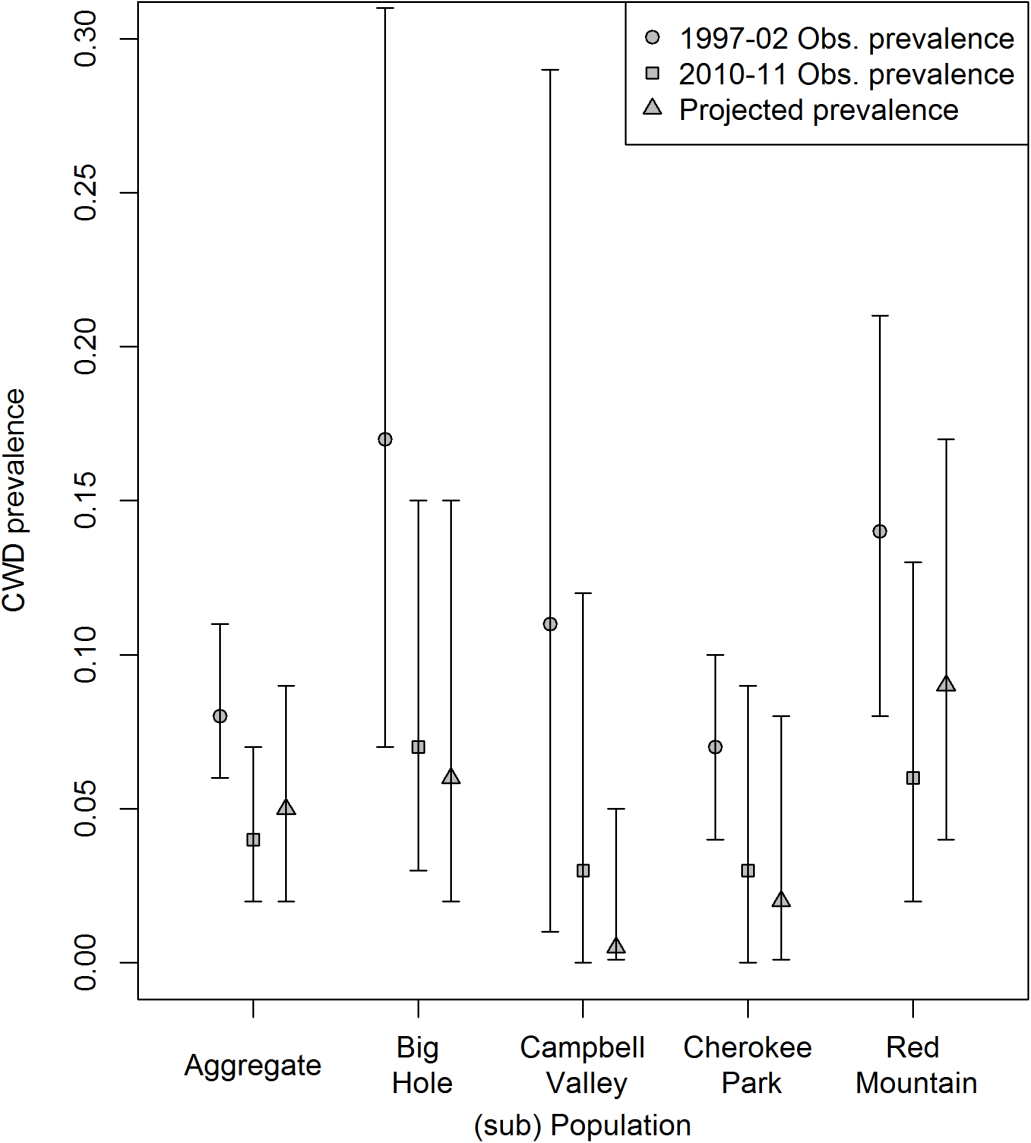
Forecasted CWD prevalence–compared to historic and recent CWD prevalence. Posterior estimates (mean and 95% equal-tailed credible intervals) of CWD prevalence during surveillance efforts during 1997–2002, capture-recapture studied deer at the time of entry into study, and forecasted short-term prevalence assuming survival, fertility, and infection conditions remained as we measured them during 2010–14. Prevalence was estimated from female deer >1 year of age.

Forecasted short-term prevalence was estimated using the survival, reproduction, and infection results from capture-recapture. Within each subpopulation and overall, the weight of evidence indicated that forecasted short-term prevalence was lower than values observed during 1997–2002 (Fig 6). Thus, current conditions likely will not lead to the higher prevalence observed during 1997–2002, suggesting a reduction in the CWD outbreak. Overall, the chance that forecasted prevalence was lower was 92%. This trend was also consistent across herds: chances that forecasted prevalence was lower than in 1997–2002 were 95% in Big Hole, 98% in Campbell Valley, 95% in Cherokee Park, and 87% in Red Mountain. Large posterior overlap of observed prevalence during 2010–11 and forecasted prevalence suggested that, if the CWD epidemic is changing under current conditions, the difference will be seen over a longer time scale than the four-year time frame of our capture-recapture research (Fig 6). Furthermore, there was some indication of gradual return to higher prevalence in Red Mountain.

## Discussion

The epidemic dynamics of CWD and its consequences for deer populations in northern Colorado and elsewhere will play out over the coming decades, well beyond the duration of any single field campaign or surveillance period. Our 5-year study was too brief to measure temporal trends. Moreover, historical data were incomplete with respect to estimating infection probability. Such challenges are commonly faced by those attempting to understand complex disease or other ecological processes that operate on protracted time scales. In this case, however, our Bayesian hierarchical approach, which integrated a matrix population and disease model with available monitoring data and process level data from contemporary capture-recapture efforts, yielded readily-interpreted estimates of key epidemic parameters despite the collective shortcomings in available data. Our approach allowed for forecasts of population and disease conditions that are far more revealing than simulation models that would traditionally be relied upon to gain such insights.

Our approach provides a framework for beginning to assess the effect of CWD on deer populations and yields parameter estimates and short term forecasts that include uncertainty via predictive process distributions. In the areas we studied, CWD did not lower population growth rate to a degree sufficient to precipitate a rapid, catastrophic decline in deer abundance. Instead, the effects on growth appear to have remained subtle despite CWD having been present for decades. Estimated growth rate under the current level of infection centered near one.

Prion infection can be thought of as accelerating the time of demise for infected individuals. Disease moves deer into demographic stage equivalent to senescence, characterized by the progressively lower survival and reproduction seen in aged individuals. Moving large numbers of young deer into "disease-related senescence" could have debilitating effects on population growth, as reflected in the dynamics of unchecked CWD epidemics described in captive deer herds [17]. However, under the regimen of natural and prescribed controls operating on herds we studied infection rates were relatively constant between 1.5 and 8.5 years of age. The average life expectancy of CWD-free deer was 5.39 (4.23−6.91) years and most (9 of 15) new infections occurred in deer ≥5.5 years old. Such timing of onset, combined with the slow progression of disease, likely muted the effects of CWD on population growth. Furthermore, infection was spatially localized: annual infection probability varied by an order of magnitude across a relatively short distance, such that overall an insufficient number of deer were becoming infected to cause rapid and widespread population collapse within the timeframe of our study.

Regardless of short-term trends and its ultimate fate, the deer population is clearly less robust with CWD. Deer abundance has declined since the 1980s (Fig 2) [27]. We cannot be sure of the ultimate cause of decline, because the deer population has been subject to changing practices to manage population demographics and CWD [27], in the face of other disease outbreaks (such as hemorrhagic disease), habitat fragmentation and a changing climate [1,28–29]. We estimated that the current population (with prevalence near 4%) is stable, and we cannot rule out slow increases or declines in abundance. Declining numbers would be the expected outcome if prevalence levels resembled those measured in this population during the late 1990s. Thus, CWD, under high prevalence, may have been an important contributing cause to the decline observed during the 1980s and 90s (Fig 6).

Managers have attempted to promote population growth by essentially ceasing female harvest since 2006 [27]. While the Cherokee Park subpopulation has responded with slow growth, small, if any, increases have been detected in the Red Mountain and Big Hole subpopulations where CWD prevalence exceeds 5%. Management of deer populations throughout the world typically includes some degree of female harvest. Even under low CWD prevalence, CWD appears to affect population growth sufficiently to make annual harvest (of females) an unsustainable management practice.

Our mark-recapture study coincided with a period of historically low deer abundance, and thus we expected higher growth rates than observed. Such discrepancy suggests that we may have underestimated CWD’s direct or indirect effects. Average adult survival for susceptible female deer was 0.82 (0.77−0.86), which centered below the range-wide average of 0.85 [20,30]. The difference between susceptible and infected deer survival was therefore less than would be expected if susceptible deer survival was nearer to or above the range-wide average, as might be expected in a low-density population.

Population growth rates in the absence of CWD were lowest in Red Mountain and Big Hole, which corresponded to highest local disease prevalence. Apparent competition is an indirect interaction among prey species mediated by a shared predator [31–32]. Mountain lions prey selectively on CWD infected deer [33] and CWD could result in an abundance of vulnerable prey, thereby enhancing mountain lion survival and reproduction [20]. A resulting outcome could be increased predation on uninfected deer and overall depression of the deer population–the inverse relationship between CWD prevalence and survival of uninfected deer supports the possibility of indirect disease effects on population growth.

The effect of CWD partly depended on the scale of analysis. When all data were combined, we did not find the remarkably high prevalence observed in two more localized deer populations (< 600 individuals) studied elsewhere. Mule deer in the Table Mesa population in northcentral Colorado exhibited ~20% prevalence among adult females with average annual infection probability of 0.23 [20]. Similarly, 42% of female white-tailed deer developed CWD during a seven year study of the Deer Creek population in central Wyoming [18]. The Table Mesa and Deer Creek populations declined and CWD was implicated as an ultimate or contributing cause [18– 20]. Annual infection probabilities approached such levels only within highly localized portions (< 50 km^2^) of our study area, and decline was the more likely scenario in associated herd units (Red Mountain and Big Hole). However, those local declines appear to be offset by growth in nearby herd units with lower infection rates. The net outcome becomes largely unchanging overall deer abundance under the conditions currently operating on these different herd units.

It follows that the long-term outcomes of CWD may depend heavily on whether high prevalence remains a localized phenomenon. CWD-causing prions appear to persist locally by transmission among individuals and from environmental reservoirs, but spatial spread almost certainly requires transport among spatially-distinct social units [16,34]. Deer show extraordinary fidelity to their female social groups and home ranges established early in life. This may limit prion mobility and thereby afford deer some natural “spatial resistance” against CWD on a larger geographic scale. However, overlap between migrant groups on summer range could bridge these social barriers, as previously suggested for the heavily infected Red Mountain herd unit [34]. Although not studied in depth here, male mule deer have larger home ranges [35], higher infection rates [20,24], and less fidelity to specific social groups [35]. Males could serve as a pathway of infection among bands of females as the males seek mates [34,35].

The long-term outcomes of CWD will depend on the extent to which this outbreak increases beyond what we observed. The weight of evidence suggests that the CWD outbreak is less severe today than during the late 1990s (and early 2000s). We cannot be sure that the 1997–2001 and 2010–2014 prevalence estimates are directly comparable. The early data were post mortem tests of harvested or culled deer and the later data were live tests of randomly chosen helicopter captured deer. Ages of sampled deer [36,37], increased vulnerability of infected deer to harvest [38], prion precursor genotype of sampled deer [39], and testing diagnostic method [40] may have influenced prevalence estimates. But, the magnitude of difference in prevalence estimates among time periods is likely greater than would be explained by sampling effects alone.

To date, we are unaware of a study that documents a decrease in CWD prevalence over time in mule deer, white-tailed deer or elk. We briefly consider three plausible explanations for our findings: a) that natural oscillations occur in CWD outbreaks; b) that the outbreak has peaked and is declining to a lower endemic level; or c) that previous management actions were more successful at suppressing the outbreak than originally believed.

Sharp & Pastor [41] illustrated that CWD outbreaks may play out as a series of reoccurring epidemics characterized by either stable limit cycles or oscillations that may dampen or amplify as a function of deer density. If this is the case, we would expect today’s declining deer population to feedback on conditions–lowering transmission rates leading to reduced CWD effects and a growing population. Increasing abundance would support higher transmission rates, deer decline, and oscillations of CWD prevalence and deer. Alternatively, Almberg et al. [21] (see also [22–24,41,42]) suggested that CWD outbreaks could reach endemic equilibrium characterized by coexistence of a smaller deer population and CWD. Under these scenarios, population prevalence would reach a lower, constant level after a period of high prevalence and deer decline.

Although neither of the foregoing scenarios can be dismissed completely, invoking them ignores the extensive management of this deer population that occurred in the years between the two time points we chose as the basis for our analyses. Management aimed to reduce CWD transmission between 2000 and 2005, which included a combination of (crude and unpopular) focal culling and a broader increase in female harvest, decreased overall deer abundance by about 25%. Analyses carried out shortly after suggested that reductions in deer density had made little impact on CWD prevalence [10]. However, our current findings suggest that these management actions may indeed have attenuated the outbreak. Observed dynamics over the last decade closely approximate those predicted from models by Wild et al. [42] that included a substantial amount of selective predation on CWD-infected individuals. That harvest could be a source of selective mortality is supported by an early notion that CWD-infected deer might be more vulnerable to harvest [43], just as infected deer also appear to be more vulnerable to vehicle collisions and predation [20,33,44]. This offers the possibility that hunting could be used as a more tightly controlled substitute for predation in studies of system responses with CWD and perhaps other similar diseases.

The protracted time-scale of the CWD outbreak is much longer than the timespan of our research, which limits our ability to identify the true explanation of our findings. Nonetheless, our research suggests that, at least for the foreseeable future (e.g., decades), mule deer populations sharing the overall survival and infection probabilities estimated from our analyses may persist but likely will not thrive where CWD becomes established as an endemic infectious disease.

## Materials and Methods

### Study Area

The Red Feather-Poudre River mule deer population is estimated ~7,300 individuals distributed across ~4,600 km^2^ of foothills and higher elevations in the northern Front Range of the Rocky Mountains, Colorado USA (Vieira 2006). Mule deer share habitats with *Cervus elaphus* (elk), *Odocoileus virginianus* (white-tailed deer) and occasionally *Alces alces* (moose), all of which may also be infected with CWD. Some of these mule deer migrate seasonally: individuals move up to 70 km between wintering areas north of Fort Collins, Colorado into the Laramie Range in southern Wyoming to the north or to the headwaters of the Poudre River to the west in north-central Colorado [34–35]. Others remain on the same range year-round. Deer occupy lands owned privately and largely used for agriculture and grazing livestock, and public lands owned and managed by the National Forest Service, the Colorado Division of Parks and Wildlife, Larimer County, and the city of Fort Collins. State and national public lands are managed for livestock grazing and to support sport hunting, which also occurs on some private lands. County and city areas are largely managed for open-space and recreation. Habitats are characterized by short grass prairie, croplands, and exurban development in the lowest elevation areas to the east and south. Foothill areas to the north and west include a variety of shrubs (e.g., *Cercocarpus* spp., *Amelanchier* spp., *Purshia* spp.) interspersed with *Pinus ponderosa* and *Juniperus scopulorum*. Higher elevation areas are characterized by *P*. *ponderosa*, *Pinus contorta*, *Psuedotsuga menziesii*, and sub alpine forests. Capture-recapture studies, CWD surveillance, and population monitoring were completed on deer wintering areas north of Fort Collins that included about 50% of the 7,300 deer in the population and 15% of the ~4,600 km^2^ area occupied by deer.

### Data Collection

We used capture-recapture methods to observe infection, survival, and reproduction of female deer during each January from 2010 to 2014. Groups of female deer were located by helicopter by searching six ~75 km^2^ areas during the initial year. One female from each group was captured. In subsequent years, deer were captured from groups residing in these same search areas. We attempted to recapture marked individuals each year.

Deer were captured by helicopter net-gun and moved to nearby processing locations. During handling, Rectal-anal mucosa associated lymphoid tissues were collected using methods described by [40] and CWD status was determined from immunohistochemistry staining. Immunohistochemistry analyses were completed at the Colorado State University Veterinary Diagnostics Laboratory (Fort Collins, Colorado, USA). We collected up to 30 ml of blood for genetic analyses. We used tooth eruption and wear patterns to estimate ages [44]. Each deer was fit with a mortality sensing collar (Advanced Telemetry Systems, Isanti, Minnesota, USA) and released. We tracked deer weekly to determine survival and approximate location. Where feasible, mortalities were investigated to determine cause of death.

Permission to work in field areas was granted by the Colorado Division (of Parks) and Wildlife, the City of Fort Collins and Laramie County, Colorado, and several private landowners. Animals handled in the field were limited to mule deer and did not involve endangered or protected species. All study animals were handled under protocols approved by Colorado State University’s Institutional Animal Care and Use Committee (11-2758A).

Long-term population and disease monitoring were completed by the Colorado Division of (Parks and) Wildlife. During December-January 2009−12, annual helicopter surveys were completed to estimate herd composition. Deer groups were located during systematic searches of known occupied areas. Encountered deer groups were classified as adult females (>12 mo), young of the year (5–6 mo), and males (>12 mo). Population density was estimated by counting deer observed on 66 established “quadrats” (0.92 km^2^, 0.25 mi^2^). Population counts were completed during 1985−89, 1993, 1996, 1998, 2000−02, and 2009. CWD surveys were done to estimate infection prevalence during 1997−2002 [45–46]. Harvested or culled deer were classified as CWD positive or negative based on IHC exam of retropharyngeal lymph node or tonsil tissue.

Using methods described in detail by [34–35] we used cluster analysis to categorize radio collared deer into wintering population units. Deer were located every 2 wk to 2 mo during November—February, 2010–14 using aerial telemetry homing techniques. Coordinate medians of winter locations for each individual were used for cluster analysis. Clusters were identified by unweighted pair-group method using arithmetic averages. We delineated the area used by radio collared deer using a bivariate kernel home range estimator. We chose the 95% use contour to represent the area commonly used by deer in winter. This region served as a boundary for helicopter surveys and disease surveillance tests. Therefore, long-term population monitoring data was restricted to a similar geographic area as our capture-mark-recapture study (S1 Appendix).

### Leslie Projection Matrix

Deer were portrayed in 14 female age and disease stages. We assumed that all deer were born uninfected and did not develop CWD during the first 6 months of life. Deer were capable of becoming infected as yearlings or during any subsequent year of life. Annual infection probability did not vary among years. We allowed different survival probabilities and fertility rates for infected and susceptible deer.

We created the vector ***n***_*t*_ to describe the number of deer in age and disease stages during January of year *t*. The first element, *n*_1,*t*_, is the number of deer that are 6 months old and CWD susceptible. The next ten elements, *n*_2,*t*_ … *n*_11,*t*_, are the number of CWD susceptible deer from 1.5 to 10.5 years old. We used three stages for infected deer to allow survival to decrease with disease progression: *n*_12,*t*_ is for newly exposed deer, *n*_13,*t*_ is for deer that survived at least one additional year post exposure, and *n*_14,*t*_ is for deer that survived at least two additional years post exposure. The vector ***n***_*t*+1_ = ***A*** × ***n***_*t*_ describes the deer population during the next year *t*+1 where ***A*** is a 14×14 projection matrix defined by,

$$\left[\begin{array}{cccccccc} 0 & f_{\text{sus},1.5} & f_{\text{sus},2.5} & \cdots & f_{\text{sus},10.5} & f_{\text{inf},0} & f_{\text{inf},1} & f_{\text{inf},2}\\ s_{\text{sus},0.5}\left(1-\psi \right) & 0 & 0 & \cdots & 0 & 0 & 0 & 0\\ 0 & s_{\text{sus},1.5}\left(1-\psi \right) & 0 & \cdots & 0 & 0 & 0 & 0\\ \vdots & \vdots & \vdots & \ddots & \vdots & \vdots & \vdots & \vdots \\ 0 & 0 & 0 & \cdots & 0 & 0 & 0 & 0\\ s_{\text{inf},0}\psi & s_{\text{inf},0}\psi & s_{\text{inf},0}\psi & \cdots & s_{\text{inf},0}\psi & 0 & 0 & 0\\ 0 & 0 & 0 & \cdots & 0 & s_{\text{inf},1} & 0 & 0\\ 0 & 0 & 0 & \cdots & 0 & 0 & s_{\text{inf},2} & 0 \end{array}\right]\;.$$ (1)

We defined time invariant infection probability as *ψ* and age-specific survival probabilities of susceptible deer as *s*_sus,0.5_…*s*_sus,10.5_. Annual survival of the oldest susceptible age was set at zero. Annual survival of infected deer during the interval that they were exposed was defined as *s*_inf,0_. Their survival during subsequent years were defined as *s*_inf,1_ and *s*_inf,2_. Annual survival during the third year post-exposure was equal to zero. Elements of the top row of ***A*** are fertilities in a Leslie matrix. To align model updates that occurred in January with the timing of the birth pulse in June, fertility elements were the product of dam survival from census to the birth pulse and recruitment–recruitment was the product of birth rate and fawn survival from birth until the next census.

We evaluated models at different spatial scales and across different geographic areas. First, we aggregated all the data to represent a single intermixing deer population. Then, because the wintering subpopulation has been used effectively to represent the spatial epidemiology of CWD in this area–likely because wintering subpopulations are known to have limited spatial overlap–we reevaluated our model for each of four wintering subpopulations at finer spatial scale.

### Bayesian Model to Estimate Matrix Parameters

#### Infection Probability

Annual infection probability was estimated from capture-recapture data (see also [46]). To do so, we needed to estimate the disease status of each individual over the course of our field study based on imperfect test results. We defined ***Z*** as an infectious status matrix, ***Z*** = [*z*_***i***,***t***_] for the *i* ^th^ deer *i* = 1…*I* and the *t* ^th^ testing occasion (year) *t* = 1…*T*. When individual *i* was infected in the *t* ^th^ year, *z*_*i*,*t*_ = 1; otherwise *z*_*i*,*t*_ = 0. The model for the initial test, *z*_*i*,1_ is described below (Eq 4). After the initial test, infection status at the current time *t* was conditioned on infection status at the previous time *t* − 1 where

$$\left[z_{i,t}|z_{i,t-1},\varphi_{i}\right]=\{\begin{array}{l} \;1,\;z_{i,t-1}=1\\ \text{Bernoulli}\left(\varphi_{i}\right),\;z_{i,t-1}=0 \end{array}.$$ (2)

We assumed that an infected individual remained infected during the subsequent testing year and a susceptible individual became infected with probability *φ*_*i*_. Infection probability was assumed to be time invariant but may have varied among individuals based on wintering population unit membership *x*_*i*_ where logit (*φ*_*i*_) = *ξ*_0_ + *ξ*_*k*_ *x*_*i*_ and *ξ*_*k*_ is the logistic model coefficient for the *k* ^th^ wintering area.

In a disease test, up to several lymphoid follicles are observed in collected tissue and the presence of infection is tested for in each follicle. We defined ***Y*** as an observation matrix, where *y*_*i*,*t*_ represents the observed number of follicles exhibiting the presence of infection. We defined the corresponding matrix, ***J***, where *J*_*i*,*t*_ is the total number of follicles obtained. False positive test results were not believed to occur. Therefore, when *z*_*i*,*t*_ = 0 then *y*_*i*,*t*_ = 0. However, we may or may not have observed at least one positive follicle when an individual was infected, meaning when *z*_*i*,*t*_ = 1, then *y*_*i*,*t*_ ≥ 0. The probability that an individual follicle was positive is *π*, and

$$\left[y_{i,t}|\pi ,J_{i,t},z_{i,t}\right]=\{\begin{array}{l} \;0,\;z_{i,t}=0\\ \text{Binomial}\left(J_{i,t},\pi \right),\;z_{i,t}=1 \end{array}.$$ (3)

The infectious status of the *i* ^th^ deer at time 1, *ψ*_0_, depended on the observed infection status *z*_*i*,1_, where a false negative was possible. That is,

$$\left[z_{i,1}|y_{i,1},\varphi_{0}\right]=\{\begin{array}{l} \;1,\;y_{i,1}\geq 1\\ \text{Bernoulli}\left(\varphi_{0}\right),\;y_{i,1}=0 \end{array}.$$ (4)

where *ψ*_0_ is the probability that an individual developed disease prior to initial testing. There is an important distinction between *ψ*_0_ and *ψ*_*i*_, since *ψ*_*i*_ only captures infection during a single year and *ψ*_0_ is the population prevalence.

We specified diffuse Beta(1,1) prior distributions for *ψ*_0_ and *π* and N(0,5) distributions for elements of ***ξ***. There were ***I*** total individuals which were in the study for a variable number of years (up to five). We defined the indicator variable *U*_*i*,*t*_ coded as zero when individual *i* was no longer in the study on occasion *t* and with the value one when the individual was in the study. Similarly we defined the indicator variable *V*_*i*,*t*_ as zero when individual *i* was not tested and with the value one when the individual was tested (Eq 7).

#### Survival

Survival probability was estimated from capture-recapture data using a hazard model. We measured survival over monthly intervals–we defined ***t*** as a vector of final time intervals after initial capture that individuals were observed. We were unable to observe the time of death for each deer, since deer died unnaturally due to hunter harvest or capture related cause, telemetry devices failed, or animals survived the extent of study. These animals were right censored.

We needed to account for the disease status of each individual and that it could change over time. A newly infected animal was likely exposed sometime between annual CWD tests. We used interval censoring and assumed that newly infected deer converted on the day of capture. Interval censoring could overestimate CWD mortality rate. But, potential bias was reduced by recapturing 85% of deer on average each year, which improved our ability to detect newly exposed animals. Also, CWD has been shown to have small effects on survival under natural infectious doses during the first year after exposure [20]. Some deer were infected prior to initial capture and including these data could further overestimate CWD mortality rate. In preliminary analyses, incorporating these individuals did not affect results and we chose to include these deer in final model runs.

Separate Cox proportional hazard functions were used for CWD infected and uninfected deer. For uninfected deer, $h(t_{i})\;=\lambda_{1}\alpha_{1}t_{i}{}^{\alpha_{1}-1}\;\text{exp}\left(\beta x_{i}\right)$ and for infected deer $h(t_{i})\;=\lambda_{2}\alpha_{2}t_{i}{}^{\alpha_{2}-1}$ where *t*_*i*_ is the final time interval that the *i* ^th^ individual was observed, ***λ*** is the hazard rate, ***α*** represents increases in hazards over time, and ***β*** are cox proportional hazard coefficients for age and wintering subpopulation. We used the variable *w*_*i*_ to indicate infected and uninfected deer, such that

$$\left[t_{i}|\alpha ,\beta ,\lambda ,w_{i}\right]=\{\begin{array}{l} \;\text{Weibul}l\left(\lambda_{2}\alpha_{2}\right),\;w_{i}=0\\ \text{Weibull}\left(\lambda_{1}\alpha_{1},\beta \right),\;w_{i}=1 \end{array}.$$ (5)

We specified N(0,1000) prior distributions for elements of log(***α***), log(***λ***), ***β*** (Eq 7).

#### Fertility

Fertility elements in our model were the product of dam survival from census to the birth pulse and recruitment. Dam survival was provided by our hazard model and recruitment was estimated from annual helicopter surveys of females and fawns. Fawns and older females were counted in groups annually. We assumed an equal sex ratio and only included half of the fawns that were counted. We defined ***y***_*f*_ and ***a*** as vectors of the total number of fawns and females observed during each annual survey. Recruitment, *r*, was estimated as the product of two ratios: the ratio of fawns to females counted at time *t* and the ratio of adults and fawns surviving *t* – 1 to *t*, to adults alive at *t* – 1 surviving to the birth pulse. It follows,

$$r=\;\frac{y_{f_{t}}}{a_{t}}\times \frac{a_{t-1}s_{\text{avg}}+y_{f_{t-1}}s_{0.5}}{a_{t-1}s_{6,\text{avg}}}\;,$$ (6)

where *s*_avg_ is adult annual survival derived using a version of our hazard model that did not differentiate age and disease, *s*_6,avg_ is adult survival over a six month interval, and *s*_0.5_ is juvenile annual survival (also derived from our hazard model). From Eq 6, we assumed that the number of fawns observed each year follows a Poisson distribution, $y_{f_{t}}\sim \text{Poisson}\;\left(r\times a_{t}\times \frac{a_{t-1}s_{6,\text{avg}}}{a_{t-1}s_{\text{avg}}+y_{f_{t-1}}s_{0.5}}\right)$. We specified a Beta(1,1) prior distribution for *r* (Eq 7).

#### Posterior Distribution

The posterior distribution and joint distributions are

$$\left[\psi_{0},\xi ,\pi ,Z,\beta ,\lambda ,\alpha ,r|Y,t,y_{f},a\right]\propto \overset{I}{\underset{i=1}{\prod }}\left({\left[y_{i,1}|\pi \right]}^{z_{i,1}}\left[z_{i,1}|\psi_{0}\right]\right)\overset{T}{\underset{t=2}{\prod }}\overset{I}{\underset{i=1}{\prod }}\left({\left[y_{i,t}|\pi \right]}^{z_{i,t}V_{i,t}}{\left[z_{i,t}|\xi \right]}^{\left(1-z_{i,t-1}\right)U_{i,t}}\right)\;\left[\psi_{0}\right]\left[\pi \right]\left[\xi \right]\;\times \;\overset{I}{\underset{i=1}{\prod }}\left({\left[t_{i}|\lambda_{1},\alpha_{1},\beta \right]}^{1-w_{i}}{\left[t_{i}|\lambda_{2},\alpha_{2}\right]}^{w_{i}}\right)\;\left[\beta \right]\left[\lambda \right]\left[\alpha \right]\;\times \;\overset{N}{\underset{n=2}{\prod }}[y_{f_{n}}|r,a_{n}\left[r\right]\;.$$ (7)

The joint posterior distribution was not available in closed formed. We used a Markov chain Monte Carlo (MCMC) algorithm to simulate from the posterior distribution and to estimate the unknown parameters of interest and the latent variables. Samples were drawn from the posterior distribution of each parameter and latent state using a hybrid Gibbs sampler. Each MCMC chain was run for 100,000 iterations and we confirmed convergence of each chain. All analyses were completed using program R [47].

### Forecasting CWD and the Deer Population

The dominant eigenvalue of our Leslie projection matrix [48] provided an estimate of the population growth rate in the presence of CWD under the conditions we observed. The dominant eigenvalue of the disease-free subcomponent of the projection matrix provided an estimate of the population growth rate in the absence of CWD. We used Monte Carlo methods to determine disease and disease-free eigenvalues, therefore, all process uncertainty associated with each model parameter was incorporated into forecasted growth rates. We use the word forecast in a narrow sense–a forecast is a predictive process distribution [49]. During each Monte Carlo iteration, we randomly chose a single value from the MCMC chain of each parameter in our Bayesian model (Eq 7). These values were used to derive the survival, infection, and fertilities inputs of our projection matrix ***A*** (Eq 1). After ***A*** was populated with inputs, we determined disease and disease-free eigenvalues and the difference between them.

Also during each Monte Carlo iteration, we forecasted population prevalence from the eigenvector associated with the dominant eigenvalue [48]. Forecasted prevalence represented what prevalence would be if survival, fertility, and infection conditions are to remain the values we measured. We compared forecasted prevalence to the posterior distribution of observed prevalence during 1997–2002 and 2010–11 to seek indication of the direction of the epidemic. Posterior distributions of observed prevalence were assumed to follow Beta (*y* + 1, *N* − *y* + 1) distributions where *y* was the number of infected deer and *N* was the number of tested deer in a sample–samples included all hunter harvested female deer > 1 year of age during 1997–2002 and all capture-recapture deer > 1 year of age at the time of initial testing during 2010–2011. At each Monte Carlo iteration, we calculated the difference between forecasted and observed prevalence–a value greater than zero would suggest an increasing epidemic, a value less than zero would indicate a declining epidemic, and a value broadly overlapping zero would indicate stationary behavior.

## Supporting Information

S1 Appendix Spatial description of capture-recapture and population monitoring data. (DOCX)

S1 Dataset Capture-recapture data and metadata file. (XLSX)

S1 Table Posterior distributions of parameters used in the Leslie matrix model. (DOCX)
