# Supplementary material for: Bayesian Modeling of Prion Disease Dynamics in Mule Deer Using Population Monitoring and Capture-Recapture Data
Source: PLoS One. 2015 Oct 28;10(10):e0140687. doi: 10.1371/journal.pone.0140687 (PMC4624844; doi:10.1371/journal.pone.0140687)
Supplement: S1 Appendix — (DOCX) [file pone.0140687.s001.docx]

**S1 Appendix: Spatial description of capture- recapture and population monitoring data**

We evaluated models representing different spatial scales and geographic areas. The first model aggregated all the data and was meant to represent a single intermixing deer population (S1.1 Figure). The remaining models only used data for the individual subpopulations (S1.2, S1.3, S1.4, S1.5 Figures). Time frames of population monitoring data collection efforts were 1985-2015 for abundance, 2009-2012 for herd composition, and 1997-2002 for CWD testing.

S1.1 Figure: The spatial overlap and extent of capture- recapture and long term population monitoring data fit to a model of the aggregate of subpopulations. The 95% use contour (bold line) delineated the area used by radio collared deer during winter. White circles show median wintering locations of radio collared deer, red outlined circles show locations of historic CWD tests, blue outlined squares show locations of quadrat surveys used to estimate population density, and green outlined squares show locations of deer groups encountered during helicopter surveys that were used to estimate population composition. White circles show median wintering locations of radio collared deer.

S1.2 Figure: The spatial overlap and extent of capture-recapture and longer-term population monitoring data fit to a model of the Cherokee subpopulation. The 95% use contour (bold line) delineated the area used by radio collared deer during winter. White circles show median wintering locations of radio collared deer, red outlined circles show locations of historic CWD tests, blue outlined squares show locations of quadrat surveys used to estimate population density, and green outlined squares show locations of deer groups encountered during helicopter surveys that were used to estimate population composition. White circles show median wintering locations of radio collared deer.

S1.3 Figure: The spatial overlap and extent of capture-recapture and longer-term population monitoring data fit to a model of the Red Mountain subpopulation. The 95% use contour (bold line) delineated the area used by radio collared deer during winter. White circles show median wintering locations of radio collared deer, red outlined circles show locations of historic CWD tests, blue outlined squares show locations of quadrat surveys used to estimate population density, and green outlined squares show locations of deer groups encountered during helicopter surveys that were used to estimate population composition. White circles show median wintering locations of radio collared deer.

S1.4 Figure: The spatial overlap and extent of capture-recapture and longer-term population monitoring data fit to a model of the Big Hole subpopulation. The 95% use contour (bold line) delineated the area used by radio collared deer during winter. White circles show median wintering locations of radio collared deer, red outlined circles show locations of historic CWD tests, blue outlined squares show locations of quadrat surveys used to estimate population density, and green outlined squares show locations of deer groups encountered during helicopter surveys that were used to estimate population composition. White circles show median wintering locations of radio collared deer.

S1.5 Figure: The spatial overlap and extent of capture-recapture and longer-term population monitoring data fit to a model of the Campbell subpopulation. The 95% use contour (bold line) delineated the area used by radio collared deer during winter. White circles show median wintering locations of radio collared deer and red outlined circles show locations of historic CWD tests. White circles show median wintering locations of radio collared deer. Insufficient data were available on population density or composition.
