# Supplementary material for: Bayesian Modeling of Prion Disease Dynamics in Mule Deer Using Population Monitoring and Capture-Recapture Data
Source: PLoS One. 2015 Oct 28;10(10):e0140687. doi: 10.1371/journal.pone.0140687 (PMC4624844; doi:10.1371/journal.pone.0140687)
Supplement: S1 Table — (DOCX) [file pone.0140687.s003.docx]

**S1 Table: Posterior distributions of parameters used in the Leslie matrix model**

**Estimates of model parameters used to populate a Leslie matrix model of a deer population chronically infected with CWD.**

|  | **Parameter**^1^ | **Mean** | **Posterior Distribution Quantiles** | | |
| --- | --- | --- | --- | --- | --- |
|  |  |  | **0.025%** | **0.500%** | **0.975%** |
| Fertility Elements | $f_{sus,1.5}$ | 0.25 | 0.20 | 0.25 | 0.29 |
|  | $f_{sus,2.5}$ | 0.25 | 0.20 | 0.25 | 0.29 |
|  | $f_{sus,3.5}$ | 0.24 | 0.20 | 0.24 | 0.29 |
|  | $f_{sus,4.5}$ | 0.24 | 0.20 | 0.24 | 0.29 |
|  | $f_{sus,5.5}$ | 0.24 | 0.20 | 0.24 | 0.29 |
|  | $f_{sus,6.5}$ | 0.23 | 0.19 | 0.23 | 0.28 |
|  | $f_{sus,7.5}$ | 0.23 | 0.19 | 0.23 | 0.28 |
|  | $f_{sus,8.5}$ | 0.22 | 0.18 | 0.22 | 0.27 |
|  | $f_{sus,9.5}$ | 0.22 | 0.18 | 0.22 | 0.27 |
|  | $f_{sus,10.5}$ | 0.21 | 0.17 | 0.21 | 0.26 |
|  | $f_{inf,0}$ | 0.22 | 0.17 | 0.22 | 0.27 |
|  | $f_{inf,1}$ | 0.17 | 0.11 | 0.17 | 0.23 |
|  | $f_{inf,2}$ | 0.11 | 0.03 | 0.11 | 0.17 |
| Survival Elements | $s_{sus,0.5}$ | 0.89 | 0.82 | 0.89 | 0.91 |
|  | $s_{sus,1.5}$ | 0.87 | 0.81 | 0.87 | 0.90 |
|  | $s_{sus,2.5}$ | 0.85 | 0.80 | 0.85 | 0.88 |
|  | $s_{sus,3.5}$ | 0.83 | 0.79 | 0.83 | 0.85 |
|  | $s_{sus,4.5}$ | 0.81 | 0.77 | 0.81 | 0.84 |
|  | $s_{sus,5.5}$ | 0.79 | 0.74 | 0.79 | 0.82 |
|  | $s_{sus,6.5}$ | 0.76 | 0.70 | 0.76 | 0.81 |
|  | $s_{sus,7.5}$ | 0.73 | 0.64 | 0.73 | 0.80 |
|  | $s_{sus,8.5}$ | 0.70 | 0.58 | 0.70 | 0.79 |
|  | $s_{sus,9.5}$ | 0.66 | 0.51 | 0.66 | 0.67 |
|  | $s_{sus,10.5}$ | 0.62 | 0.43 | 0.62 | 0.63 |
|  | $s_{inf,0}$ | 0.68 | 0.50 | 0.68 | 0.84 |
|  | $s_{inf,1}$ | 0.41 | 0.19 | 0.41 | 0.64 |
|  | $s_{inf,2}$ | 0.18 | 0.02 | 0.18 | 0.43 |
| Infection | $\psi$ | 0.04 | 0.02 | 0.04 | 0.07 |

^1^ Model parameters are defined in the methods section of the main body of manuscript text.
